# What may encourage or deter health services utilization by people living with or at the risk of HIV/AIDS in special health centers? Qualitative evidence from a stigmatized community

**DOI:** 10.1186/s12889-024-18480-3

**Published:** 2024-04-08

**Authors:** Mohammad Bazyar, Samaneh Tahmasebi Ghorabi, Jamil Sadeghifar, Mohammad Ranjbar, Reza Pakzad, Fatemeh Bonyadi, Keyvan Khasi, Ebrahim Shakiba, Mahtab Nourbakhsh, Leila Rezeghian, Boshra Noshadi, Mehrdad Bavandpour, Azim HasanBeigi, Anahita Behzadi

**Affiliations:** 1 Health Management and Economics Department, Faculty of Health, Ilam University of Medical Sciences, Ilam, Iran; 2 Clinical Research Development Unit, Emam Khomeini Hospital, Ilam University of Medical Sciences, Ilam, Iran; 3 Health and Environment Research Center, Ilam University of Medical Sciences, Ilam, Iran; 4 Health Policy & Management Research Center, Department of Health Management and Economics, School of Public Health, Shahid Sadoughi University of Medical sciences, Yazd, Iran; 5 Department of Epidemiology, Faculty of Health, Ilam University of Medical Sciences, Ilam, Iran; 6 Department of Public Health, Faculty of Health, Ilam University of Medical Sciences, Ilam, Iran; 7 Behavioral Diseases Research Center, Public Health Deputy, Kermanshah University of Medical Sciences, Kermanshah, Iran; 8 Communicable Diseases Department, Public Health Deputy, Ilam University of Medical Sciences, Ilam,, Iran; 9 Health Services Management Research Center, Institute for Futures Studies in Health, Kerman University of Medical Sciences, Kerman, Iran; 10 Department of Health Management, Policy and Economics, Faculty of Management and Medical Informatics Sciences, Kerman University of Medical Sciences, Kerman, Iran

**Keywords:** HIV, AIDS, Iran, Behavioral diseases Counseling centers, Vulnerable women’s Counseling centers, Facilitators

## Abstract

**Background:**

Behavioral Diseases Counseling Centers (BDCCs) and Vulnerable Women’s Counseling Centers (VWCCs) in Iran are the main peripheral centers that offer educational, counseling, diagnostic, preventive, curative and protective services to individuals living with or at high risk of contracting HIV/AIDS and female sex workers respectively. Due to the social stigma surrounding HIV in Iran, this study aims to identify the factors that may hinder or encourage HIV/AIDS patients and women with risky sexual behaviors from visiting these centers.

**Methods:**

Conducted in 2023, this qualitative study involved individuals visiting BDCCs and VWCCs in two western provinces of Iran, Ilam and Kermanshah. The study participants included 21 health staff members working in BDCCs and VWCCs and 20 HIV/AIDS patients and vulnerable women with unsafe sexual behaviors referring to these centers. Purposive, snowball and maximum variation sampling techniques were applied to interview the participants. Interviews were conducted between January 5th and May 21st, 2023, using a semi-structure guideline. Interviews were transcribed and content analysis approach was applied to analyze data using MAXQDA20 software.

**Results:**

According to the findings, the barriers and facilitators of visiting specialized centers for HIV/AIDS patients and vulnerable women were categorized into three main categories, 10 subcategories and 35 sub-subcategories including: Medical and operational processes (4 subcategories and 12 sub-subcategories), mutual interactions between the personnel and visitors (people living with and at the risk of getting HIV/AIDS) (3 subcategory and 13 sub-subcategories), and physical characteristics of the centers (3 subcategories and 10 sub-subcategories).

**Conclusions:**

To improve the performance of BDCCs and VWCCs and encourage people living with and at the risk of contracting HIV/AIDS to visit these centers regularly, health policy makers should consider modifying clinical processes, physical features, personnel behaviors and visitors’ concerns raised by the interviewees and the issues identified in this study.

## Background

According to the latest report of the Joint United Nations Program on HIV/AIDS (UNAIDS), in 2019, 38.0 million people globally were living with HIV, and around 690,000 people died from AIDS-related illnesses [1]. This threat is dominant for countries in East Mediterranean Region, such as Iran [2]. In 2019, 59,000 [33,000–130,000] adults and children were living with HIV in Iran. The number of men aged 15 and over living with HIV was estimated at 43,000 [23,000– 91,000], while that of women stood at 16,000 [8800–32,000] [3, 4]. Injection drug use is considered the main cause of HIV transmission in Iran [5]. According to a previous study, there were about 230.000 people injecting drug in Iran in 2016 [6].

Since finding the first case of HIV/AIDS in the country, Iran has established different national and provincial institutions and has implemented different programs aiming to put the disease under control. For example, in 2003, the Supreme Council for HIV/AIDS Prevention Planning (SCHAPP) was formed [7–9]. Accordingly, at national and provincial level, the department for Controlling Communicable Diseases was created within Ministry of Health and Public Health Deputy of Medical Universities. Besides, at district level, the peripheral institutions to provide specialized health services for HIV/AIDS patients are called “Behavioral Diseases Counseling Centers” (BDCCs). These centers are governmental and operate as a part of District Health Network under supervision of Public Health Deputy. BDCCs provide educational, counseling, diagnostic, preventive, and curative services to those who live with or are at the high risk of contracting HIV/AIDS [6, 10, 11]. Following groups can use the services of BDCCs: people who inject drugs; individuals with sexually transmitted diseases; people with risky sexual behaviors like homosexuals and vulnerable women with unsafe sexual behaviors; health workers and other professions who are in contact with high risk groups or are the risk of encountering sharp and infectious tools; and also individuals looking for counseling services. A group of health staff including infectious diseases specialist, psychologist, general physician, public health technician and midwifes work in these centers.

However, emerging evidence indicates that vulnerable women and female having uncontrolled and unsafe sex behaviors are becoming increasingly a leading source of HIV incidence [12]. For this reason, female sex workers are considered the second most at risk population for HIV transmission [13]. As prostitution and sex work are illegal and extremely stigmatized in Iran [14], it has affected the process of diagnosis and treatment of HIV/AIDS adversely and consequently has posed major challenges to cope with it in Iran [5, 15]. To encourage safe sexual behaviors and control spread of HIV/AIDS in this part of population, other centers so-called “Vulnerable Women’s Counseling Centers” were created separately to provide counseling services, and distribute preventive and protective services for female at the risk of getting HIV (women live with HIV/AIDS use their curative services from BDCCs).

Additionally, several other supportive institutions such as “Drop in Centers”, “Outreach Team”, “Methadone Maintenance Therapy”, “Hot Lines”, and “Positive Clubs” have been established which provide supportive and empowering services for addicted, unprivileged and marginalized groups [9]. However, Iran is still struggling reaching national and international health indicators regarding HIV/AIDS control. For example, the reduction of HIV/AIDS cases in Iran is lower than the global rate. Since 2010, the worldwide reduction in new HIV infections was 18%, while it was 10% for Iran [16, 17]. Other indicators such as the rate of adherence to antiretroviral therapy (ART) which are crucial for successful HIV treatment shows that the situation is not promising. For example, the rate of ART regimens among Iranian people living with HIV/AIDS (PLWHA) is so as low as 20% and only 17% are ART adherent and virally suppressed [5].

Various factors can hinder appropriate service delivery to people living with HIV/AIDS which in turn can impede implementing health program for HIV/AIDS, including diagnosis of new cases and adherence to treatment. Multiple structural, social, and psychological challenges contribute to non-adherence to treatment which stems from different causes [18–20]. Among the most robust predictors of ART non-adherence is social stigmatization of HIV [18]. HIV stigma affects working on HIV/AIDS objectives adversely and undermine all phases of health care provision from late diagnoses of HIV by discouraging high risk individuals from taking test [21, 22], to suppressing antiretroviral treatment (ART) coverage and viral suppression rates as it impedes individuals from looking for the healthcare services they need and from attending medical appointments and taking their medication [23, 24]. Other findings from various contexts suggest that different factors related to the health centers and health personnel may hinder HIV/AIDS individuals from accessing services. These include concerns about lack of privacy and confidentiality during HIV testing, distrust of healthcare workers’ ability to keep personal information confidential, stigma associated with being seen at health services, fear of punishment or criminalization, transportation costs, and financial barriers. These barriers and facilitators vary across different populations and settings, highlighting the need for more studies to identify other context based deterrents and facilitators [25–30].

Previous studies in Iran have addressed other aspects of health status of people living with HIV/AIDS such as antiretroviral therapy adherence and determining factors in general [5, 31], their quality of life [32–34], factors affecting their survival [35] and late diagnosis [36], and etc. However, to the authors’ best knowledge, no study has still been carried out specifically on Behavioral Diseases Counseling Centers and Vulnerable Women’s Counseling Centers in Iran to reveal what may hinder or encourage people living with and at the risk of getting HIV/AIDS coming to these centers at the first step as they are the main peripheral centers that should attract people to take test, get their treatment, receive medications and other protective and preventive services, follow-up their health status, and adhere to treatment for the rest of their life. Conducting this study in Iran, as an Islamic country with strict laws and heavy social stigma against HIV/AIDS and sexual promiscuity, can provide new insights and reveal hidden angles on barriers to accessing services specialized for populations at the risk of HIV/AIDS that can be applicable to other countries with similar constrains.

## Method

### Study setting

This article presents the findings of qualitative section of a larger mix-method study done in 2023. The study population consisted of everyone visiting Behavioral Diseases Counseling (BDCCs) and Vulnerable Women’ Counseling Centers (VWCCs) in two western provinces in Iran, namely Ilam and Kermanshah, in 2023. These two regions were selected as they generally have similar cultural and social context and the members of research team are from these two provinces. Another reason to mention is that Ilam province (the initial place for the purpose of the study) is a small region and the number of patients to participate was not enough to cover both qualitative and quantitative phases of the main study, so it was decided to extend the study to the neighboring metropolis of Kermanshah aiming to have adequate number of participants to run the study (The distance between these two cities is about 170 km). Although this decision enriched findings of the study because the BDCCs and VWCCs of these cities are located in different geographical regions and there are potential differences among them in terms of physical and structural conditions of the centers, service provision processes, staff performance, and etc. These differences provided a better opportunity to extract the most factors affecting the clients’ trust as possible. Kermanshah has two BDCCs and two VWCCs and Ilam has only one BDCC. All of these five centers were selected to recruit interviewees. For better understanding of the health facilities as a part of the study context, the number and composition of health staff working in these centers in 2023 are shown in Table 1.

**Table 1:** The number and composition of health personnel working in facilities selected for the purpose of study

University	Center	Number of personnel	Occupation (number)
Ilam University of Medical Sciences	Behavioral Diseases Counseling Center	8	Midwife [1] Psychologist [1] General physician [1] Infectious disease specialist (1, part time) Clinical laboratory technician [1] Head of the center (public health expert) [1] Janitor (1, part time) Social worker [1]
Kermanshah University of Medical Sciences	Behavioral Diseases Counseling Center, number 1	15	Midwife [1] Psychologist [2] Pharmaceutical technician [1] Psychiatrist (1, part time) General physician [3] Infectious disease specialist [1] Pediatric specialist (1, part time) Clinical laboratory technician [2] Head of the center (Ph.D of health promotion) [1] Receptionist [1] Janitor [1]
	Behavioral Diseases Counseling Center, number 2	8	Midwife [1] Psychologist [1] Pharmaceutical technician [1] General physician [2] Clinical laboratory technician [1] Head of the center (public health expert) ( [1] Receptionist and Janitor [1]
	Vulnerable Women’s Counseling Centers, number 1	2	Midwife [1] Psychologist [1]
	Vulnerable Women’s Counseling Centers, number 2	3	Midwife [2] Psychologist [1]

-Number of active and inactive HIV/AIDS patients and female sex workers were not provided for confidentiality reasons.

### Sample size, sampling method, and interview guide

The study population composed of two main groups: (1) HIV/AIDS patients and people visiting BDCCs, and vulnerable women coming to VWCCs, (2) the health staff members working in these centers. So purposive sampling was applied to select interviewees from the above groups.

Moreover, since the researchers were not familiar with all the knowledgeable people at the beginning of the study, further samples were identified using snowball sampling. To do so, at the end of each interview, the participants (mainly health workers) were asked to introduce other people with relevant knowledge and experience for interview, even if they worked in other organizations. Using snowball sampling, two experts from other organizations working with vulnerable groups such as the State Welfare Organization (Sazman-e Behzisti) and two experts with previous experience in DBCCs were identified. Furthermore, we tried to apply maximum variation sampling by selecting interviewees from 5 BDCCs and VWCCs located in different geographical regions; interviewing people from different groups including health staffs with different professions from different organizations, people living with HIV/AIDS and vulnerable women; and recruiting interviewees from both sexes. Apart from old patients, we tried to interview new cases if possible. The sample size is directly associated with data saturation and interviews continued until no new data or idea was revealed, and interviewees repeat the subjects already mentioned. A semi-structured qualitative interview guide was used to do the interviews. The questions were based on the study objectives. The initial version of the questions was approved through discussions among the research team members and feedback that we get from the key informants (health workers working BDCCs and VWCCs) during the first interviews. These were the main questions outlined in the interview guide: What were your initial concerns when considering a visit to the center? What about the next visits? Which attributes of the healthcare staff motivated or dissuaded you from continuing to visit the center? What aspects of the clinical and administrative procedures instilled confidence or apprehension in you regarding the utilization of healthcare services at the center? What is your assessment of the center’s location and infrastructure? What precautions do you take to address any concerns prior to visiting the center? While the main questions remained consistent, additional questions were introduced in subsequent interviews as new ideas and inquiries emerged throughout the research process. It is important to highlight that unlike quantitative studies, qualitative research allows for flexibility in the interview guide, enabling researchers to adapt and refine questions as they deepen their understanding of the topic over the course of the study.

### Individual interviews and focus group discussions

The interviews were initiated after official correspondence with the Public Health Deputies of Ilam and Kermanshah Medical Universities. An interview guide was used to conduct the interviews. The interviews normally began with simple and general subjects and moved toward more specific questions. Also, probing questions were employed to obtain more accurate and in-depth information by encouraging the interviewees to give more explanations. At the beginning of each interview, after explaining the importance of recording the interview and assuring them of the confidentiality of the contents, the interview was recorded using two voice recorders. At the end of each interview, the interviewee was asked to introduce experts and people with knowledge in the field of this study to be interviewed to gain complementary information. The main researcher (MB), interviewed all health staff members from Kermanshah and Ilam. Whenever possible, these interviews were conducted face to face at the BDCCs and VWCCs. In 8 cases, personnel from Kermanshah were interviewed over the phone due to physical distance. The phone interviews were conducted at home in privacy, with the speakerphone on and the audio recorded using a recorder. In-depth individual interviews were utilized in, 14 cases, involving a range of health professionals such as general physicians, psychologists, midwives, laboratory technicians, and public health experts. Table 2 displays key characteristics of the health staff interviewed, including their position, gender, age, work experience, years in the field of HIV/AIDS and the duration of the interviews. There was no repeated long interviews but we asked the initial interviewees additional questions in-person or on the phone later on during the research as new questions arose. In two cases, despite multiple follow-ups and setting time to interview over phone, we were unable to conduct interviews due to the busy schedules of the interviewees. To foster deeper discussions and extract more nuanced insights about the subject, focus group discussions (FGDs) were employed whenever possible. Two FGDs were conducted, one with four health staff members at the Ilam BDCC and another with three employees at BDCC number 1 in Kermanshah. These FGDs took place in the psychologist’s room at midday to ensure a quiet and conducive environment for open dialogue without interruptions. The main investigator (MB) facilitated the discussions, each lasting for an hour. The use of group discussions created an interactive setting that encouraged participants to recall and share additional details regarding the factors influencing people’s trust in visiting the BDCCs and VWCCs. In total, 21 personnel were engaged in the study, with 14 individuals participating in in-depth individual interviews and 7 taking part in FGDs.

**Table 2:** The main characteristics of health personnel interviewed

Total number of health personnel interviewed	Position of health personnel (number of interviewees)	Gender of health personnel (number of interviewees)	Minimum, average, and maximum age of health personnel interviewed (years)	Minimum, average, and maximum work experience of health personnel interviewed (years)	Minimum, average, and maximum work experience of personnel in the field of HIV/AIDS (years)	Minimum, average, and maximum length of interviews with personnel (minutes)
21	General practitioner [2] Psychologist [8] Midwife [6] Laboratory technician [1] Public health technician [3] Social worker [1]	Male [6] Female [15]	32, 46, 61	1, 19, 27	1, 6, 20	30, 45, 70

However in regard with the HIV/AIDS patients and women coming to the BDCCs and VWCCs, another approach was applied. In accordance to confidentiality principles, one of the staff members from each center, was selected to conduct the interviews on behalf of the research team. To do so, three health workers from three centers were selected, BDCC number 2 and VWCC number 2 from Kermanshah and the only BDCC from Ilam. All of these health staff who agreed to collaborate were female. In other centers, no health staff agreed to participate in doing the interviews. Having a close relationship with patients and being interested in research project were the criteria to select the interviewers. The selected health staff were interviewed as key informants which enabled them to gain a thorough understanding of the research objectives and qualitative interview techniques. Moreover, the selected members were trained about the main ethical principles and how to conduct interviews. Additionally, the recorded interviews conducted by the health staff were heard and analyzed several times by the main investigator (BM). Constructive feedback was provided to the interviewers to improve the quality of subsequent interviews. Notably two of these staff held Master of Science degrees in relevant health fields and possessed a solid understanding of fundamental health research methodologies. In total, health staff could interview 20 HIV/AIDS patients and female sex workers, including 6 individuals from Ilam BDCC, 12 and 6 individuals from Kermanshah BDCCs and VWCCs respectively. Patients were mainly male and the interviews lasted from 10 min to 35 min (20 min on average). The duration of interviews with patients was normal as they had specific and limited but different concerns for visiting or not visiting health centers. On the other hand, one of the limitations of the interviews was that some of participants wanted to finish the interview quickly and leave the center. Only three of cases in BDCCs were new visitors while the rest were regular and routine visitors of the BDCCs and VWCCs. In 8 cases, mainly female sex workers, they did not allowed recording their voice, so interviewers took notes of the main points. Furthermore, according to the interviewers, approximately 7 visitors refused to participate in the study due to inappropriate mental conditions, being in rush, and fear of their identity being revealed by recording their voices. Interviews were done within the time period between January 5th and May 21st, 2023. For the confidentiality purposes, no detailed demographic information of the patients was provided. In both groups (personnel and visitors), we reached saturation at about 17th interviewee, but we conducted 6 extra interviews, 3 for each group, to ensure not missing new data. Literature review was done from 10 July 2022 to 25 June 2023, no time limitation was applied for extracting the related articles.

## Ethical issues

This study was approved by Ethics Committee of Ilam University of Medical Sciences. Also, a reference letter was provided by the Ethics Committee for the Security Departments, and Infectious Diseases Departments of Health Deputies of Ilam and Kermanshah Medical Universities, and their approvals to start the project were obtained. Moreover, research team were introduced by Infectious Diseases Departments in both universities to the BDCCs and VWCCs for doing interviews. Considering the sensitivity of the subject and the necessity of observing all ethical issues, the required permissions were also obtained from the Infectious Disease Control Center and the HIV office of the Health Deputy of the Iranian Ministry of Health and Medical Education. In addition, in line with confidentiality principles, the research team were not allowed to interview the HIV/AIDS patients and women coming to BDCCs and VWCCs directly. To do so, one of the staff members from each center, preferably the counselor, was selected to conduct the interviews.

## Qualitative data analysis

Each interview was transcribed and analyzed immediately before doing the next one. This contributed to the conduction of later interviews as the researchers were able to have a more comprehensive view of the subject under studying. One author (MB) initially analyzed and indexed the transcribed interviews. Content analysis was used to analyze the interviews according to the six-step framework of Braun and Clarke including familiarizing with data, generating initial coding, searching for themes, reviewing of themes, defining and naming of themes, and reporting the findings [37]. Using inductive approach, we categorized the emerging codes according to the patterns and similarities between them. The categories were generated from the patterns within the codes, frequency and repetition, and their importance to the participants. In the next step, categories were further grouped into more general categories according to the research objectives. The emerging categories and sub-categories, appropriateness of titles chosen for them, and choosing right category for each sub-categories were constantly discussed among the research team and amendments were made whenever necessary. The codes were classified into three layers including categories, subcategories, and sub-subcategories. The final classification was shown to the health staff members in Ilam BDCC once again and few further codes were identified. All the processes related to data coding and emerging themes were carried out using MAXQDA20 software (VERBI Software. MAXQDA20. Berlin: VERBI Software, 2020).

### Trustworthiness

To ensure the trustworthiness of our findings, we adhered to four criteria outlined by Lincoln and Guba (1986) which encompass credibility, transferability, dependability, and confirmability [38, 39]. “Credibility”, focusing on the question of how well the findings reflect reality, was achieved through the following techniques. The primary researcher (MB) spent nearly 4 months constantly reviewing and analyzing qualitative data to enhance credibility. Additionally, member-checking validation was utilized by sharing initial categories and sub-categories with the healthcare staff at Ilam BDCC. During interviews, clarifying questions such as ‘What do you mean?’ or “do you mean….?” were posed to address any ambiguity in responses, ensuring a clear understanding of the interviewees’ intentions. Feedback was provided during interviews to confirm mutual understanding and interpretation between interviewers and interviewees. Peer review was employed as a method to enhance credibility through ongoing discussions between two authors (MB and JS) regarding the accuracy and categorization of emerging categories and sub-categories. Negative cases were also highlighted to showcase instances where there was a discrepancy in perspectives among interviewees, further bolstering credibility. To ensure the findings’ applicability to other settings, the researchers employed purposive and maximum variation sampling in line with the concept of “transferability.” Furthermore, the technique of “thick description” was utilized to offer a comprehensive portrayal of the context (including the HIV/AIDS situation in Iran, the cities of Ilam and Kermanshah, and the characteristics of the healthcare personnel in the selected facilities) to bolster transferability. Furthermore, we implemented an audit trail by meticulously detailing and documenting each step of the research process, along with explaining the rationale behind the research decisions made at various stages of the study, in order to guarantee “dependability”. Additionally, we utilized “peer debriefing” to enhance dependability by engaging a colleague who works in the HIV/AIDS field in Ahwaz as an external auditor to review and provide feedback on the finalized classification of codes. “Confirmability”, the final criterion for trustworthiness, focuses on ensuring that “the researcher’s interpretations and findings are clearly rooted in the data, necessitating the researcher to demonstrate how conclusions and interpretations were reached.” To achieve this, we employed methodological triangulation and included quotations. For example, we conducted in-depth individual interviews, focus group discussions and field notes concurrently to enrich data collection. The primary researcher diligently recorded field notes before and during the research process to capture every relevant phenomenon, such as observations, conversations, personal experiences, emerging thoughts, insights gained, and new questions that arose during the study. For example, notes taken during visits to the research sites and informal discussions with healthcare staff yielded valuable insights that prompted further inquiry into the topic. Furthermore, we included quotations from patients and healthcare staff members to confirm that the findings were derived directly from the data [38, 40–43].

## Results

According to the views of the interviewees, at the open coding phase, about 600 codes were identified. These codes were categorized into three main categories, 10 sub-categories and 35 sub-subcategories including: Medical and operational processes (4 subcategories, 12 sub-subcategories), Mutual interactions between personnel and visitors (people living with and at the risk of getting HIV/AIDS) (3 subcategory, 13 sub-subcategories), and Physical characteristics of the centers (3 subcategories, 10 sub-subcategories)(see Table 3).

**Table 3:** Classification of the deterrents and facilitators affecting health care utilization by HIV/AIDS people and women with risky sexual behaviors in Behavioral Diseases Counseling (BDCCs) and Vulnerable Women’s Counseling Centers (VWCCs) in Iran

Category	Subcategory	Sub-subcategory
**Medical and operational processes**	**Counseling services considerations**	Counseling the clients even before admission and administrative operations on their first visit to BDCCs and VWCCs
		Counseling in a room with the doors closed to keep the client calm
	**Identity information considerations**	Not insisting on receiving identity information of HIV/AIDS patients and sex workers
		Considering the fear of patient regarding disclosure of their disease and the importance of following confidentiality principles by the personnel
		Requirements of storing identity information of HIV/AIDS and sex workers and how to keep them safe
	**Providing remote services for patients**	Reaching health services to HIV/AIDS patients without coming to BDCCs and VWCCs in person as much as possible
		Considerations and requirements of using phone for follow-ups
	**Enhancement and diversification of services and reducing social stigma**	Providing services, regardless of the cause of the HIV/AIDS, and not focusing on the real cause
		Diversifying the range of diseases covered by the BDCCs, not only HIV/AIDS patients
		Providing complementary specialized diagnostic tests and supportive non-medical services
		Avoiding provision of specialized health services in specific weekdays and preventing gathering of patients in the BDCCs
		Giving patients hope of having a normal life and ensuring them of the effectiveness of the medication and treatment
**Mutual interactions between personnel and visitors (people living with and at the risk of getting HIV/AIDS)**	**Personnel behavior**	Not judging and reproaching HIV/AIDS patients and sex workers
		Having normal and friendly behavior with HIV/AIDS patients
		Previous patients satisfied with and trusted in BDCCs and VWCCs as a key factor to assure new cases to visit these centers
		Training the personnel regarding the principles of interaction with HIV/AIDS patients
		Getting a better physical and mental feeling in each visit
		Wearing a formal uniform
	**Concerns of visitors**	Patients’ concerns about seeing familiar personnel
		Concerning about legal problems
		Avoiding changing the personnel working in BDCCs and VWCCs
		Concerns about taking medications
	**Precautions by visitors**	Excuses and tricks of patients and vulnerable women in their first visit
		Using disguise and protective equipment to remain anonymous
		Going to the BDCCs and VWCCs during the last working hours: Spending the minimum possible time in the center
**Physical characteristics of centers**	**Location of BDCCs and VWCCs**	Area of operation: in a quiet or crowded place?
		Working as separate and independent BDCCs and VWCCs as or as a part of a larger building?
		Establishing fixed centers and not changing the location BDCCs and VWCCs
		Physical space (size) of BDCCs and VWCCs
	**Geographical distribution of BDCCs and VWCCs**	Physical distance between BDCCs and VWCCs and living place of people
		Not having a catchment area and no geographical division for BDCCs and VWCCs
		Increasing the number of BDCCs and VWCCs
	**Physical evidence**	The sign board of the BDCCs and VWCCs
		Not having surveillance cameras installed
		Not using signs of HIV/AIDS in the BDCCs

### Medical and operational processes

#### Counseling services considerations

##### Counseling the clients even before admission and administrative operations on their first visit to BDCCs and VWCCs

Counseling clients before admission and administrative operations on their first visit to BDCCs and VWCCs is crucial. Interviewees suggested guiding patients directly to the counseling room without any questions or admission processes, allowing them to receive consultation services immediately. It was also recommended to have the counseling room located near the entrance to minimize contact with the personnel or other patients, reducing concerns and increasing their trust in the center’s services.

*“” When a person comes to the center for the first time, it is better not request for any demographic information, instead he or she should be referred directly to the counselor’s room” (One of the psychologist in BDCCs)*.

##### counseling in a room with the doors closed to keep the client calm

Conducting counseling in a closed room or with the door partly open (if the counselor and the client are not of the same sex or if the client requests it) is important for HIV/AIDS patients in DBCCs and vulnerable women in VWCCs. This ensures confidentiality during conversations. They also highlighted that to maintain the patient’s calmness, other personnel should avoid entering the room unless necessary.

*“Another reason I trust you (female psychologist in one of the BDCCs) was that you closed the door and I felt secure that no one can hear my statements” (a young man looking for HIV/AIDS test on his first visit)*.

#### Identity information considerations

##### Not insisting on receiving identification information of HIV/AIDS patients and sex workers

Given the social stigma associated with AIDS, one of the most frequently mentioned statements by interviewees was that the centers must not insist on receiving identity information at least in first visits, including first and last name, national ID number, address, age, etc. In addition, the patients are even encouraged to use nicknames instead of providing personal information. This helps build trust between patients and the health staff, and patients may be willing to provide their real information in subsequent visits once they trust the center. Interviewees also mentioned that no notes should be taken during the first counseling sessions, and personnel should not enter patient information into the computer simultaneously, further building patient trust.

*“They resist giving the right information about themselves. For example they don’t give their telephone number or their numbers are wrong. The addresses they give are not precise at all and it is not possible for us to follow up or send reminder” (one of the health workforces from VWCCs)*.

##### Considering the fear of patient regarding disclosure of their disease and the importance of following confidentiality principles by the personnel

Maintaining confidentiality is crucial to address patient fears regarding the disclosure of their disease. Personnel should emphasize confidentiality and assure patients that their information will not be shared with anyone, including their closest family members, without their permission. In cases where patients and personnel know each other, clear assurances should be given regarding confidentiality. Personnel should also try to avoid drawing attention to familiar clients and should refrain from calling patients by their names to uphold confidentiality principles.

*“Some patients may come at the same time, and we assign them to different rooms. We try to refer one of them to the doctor’s office, one to the psychologist’s, and another to other wards so they do not see each other. For instance, two of them might be from the same city, so they are afraid of being recognized.” (One of the midwives in BDCCs with 6 years’ work experience in HIV/AIDS)*.

Moreover, some of the patients suggested that if they need health services in hospitals or other medical centers, their companions must not be involved in the treatment process. For instance, their companion should not be asked to get the patient’s test results, and everything should be done by the personnel in order not to increase the risk of the disease being revealed to their families. In this regard, some of the patients believed that if the HIV/AIDS test results are positive, no one else must be informed, and if needed, their families or relatives should be told that the results were negative.

##### Requirements of storing identity information of HIV/AIDS patients and sex workers and how to keep them safe

In BDCCs, each patient is assigned a code for their medical procedures. However, patients who give their real identity information, such as their national code or the cause of the disease, are worried about who may get access to this information. To address these concerns, some centers have implemented measures to improve information security. For example, only select staff members, such as the doctor or counselor, can have access to patients’ files and in some cases, on patients’ request, other staff members only have access to the necessary information (e.g., patients requiring special treatment without disclosing the cause of their disease). Patients also expressed concerns about storage of their information, and the possibility of it being revealed if the computers are hacked.

*“No one else except the health forces working here (BDCC) should access to our files and the computer. It is risky to store the identity information of patients in the computer. There are people who are intelligent and can access to our information by hacking the computer” (A regular old male HIV/AIDS patient)*.

#### Providing remote services for patients

##### Reaching health services to HIV/AIDS patients without coming to BDCCs and VWCCs in person as much as possible

In an effort to improve the services and gain patients’ trust, BDCCs have implemented measures to reduce unnecessary in-person visits while maintaining confidentiality and medical protocols. For example, on the patient’s request, tests can be performed outside the center such as in the car, during the first visits. Some patients may also prefer to receive their medication outside the center or for a longer period of time. In certain cases, the medication can be posted to patients’ addresses, or trusted individuals, such as friends or family members, can be designated to receive medication.

*“I remember one of new visitors asked us to take a test from her in her car without registering her name”. (A male counselor) (A male counselor with 6 years’ work experience in the field of HIV/AIDS)*.

##### Consideration and requirements of using phone for follow-ups

Another important consideration continuing treatment is the requirements and sensitivities surrounding phone calls to contact patients. According to the interviewees, many patients initially refuse to provide their phone numbers on first visits and strongly resist follow-up phone calls due to concerns about unintentionally revealing their disease to their families. As a result, patients are only willing to give their phone numbers if they trust the center and if personnel take necessary precautions when making calls. For example, personnel can phone specified family members if the patients is not accessible, or they can introduce themselves as calling from the health center rather than the BDCCs. Patients strongly disagree with the centers giving their phone numbers to other governmental institutions without prior permission. Interviewees suggested that only one personnel member should make contact with patients for follow-up to help patients feel secure. Some patients, particularly those who have been in contact with the center for a longer period or those who are less conservative, were satisfied with the follow-up calls and considered them a positive sign of the personnel’s commitment. Having an active phone line in the center can help patients receive the services more conveniently and securely as they can call the center and m check for any potential acquaintances before going.

*“Some of the patients call before coming to the center. Some of them have my own number and call me directly and ask if the center is crowded or quiet. Although patients’ having my number increases my work labor but I believe this has been effective in trusting the center” (A male counselor in BDCCs with 6 years’ work experience in the field of HIV/AIDS)*.

*“I wanted to call before visiting the BDCC because it was my first time coming to these kinds of centers, but I could not find any telephone number” (A young male HIV/AIDS patient)*.

#### Enhancement and diversification of services and reducing social stigma

##### Providing services, regardless of the cause of the HIV/AIDS, and not focusing on the real cause

According to the interviewees, usually during the first visits, health staff members do not focus on the cause of HIV/AIDS and provide services without insisting on knowing the real cause. Sometimes, morally acceptable causes of HIV/AIDS may be reported to family members to protect patients’ dignity. Patients reveal the real cause when they have established trust in the center and the personnel’s confidentiality. In some cases, an unreal cause for HIV/AIDS is recorded in patients’ file at their request to further gain their trust.

##### Diversifying the range of diseases covered by the BDCCs, not only HIV/AIDS patients

One thing that causes patients to be cautious about visiting these centers in-person is that the **BDCCs** provide services exclusively to HIV patients, and this increases patients’ concerns about visiting these centers. The interviewees, both the personnel and the patients, suggested that health services for other related diseases such as hepatitis, HPV, and other blood diseases should be provided so that patients can visit the centers with better excuses.

*“I wish we could also cover other diseases like HPV or Hepatitis B, so the patients can say “I am here for Hepatitis B if they see someone familiar in the center. We are working exclusively on HIV now and it is one of the drawbacks of our BDCC” (A female general practitioner with 4 years’ work experience in HIV/AIDS)*.

##### Providing complementary specialized diagnostic tests and supportive non-medical services

According to one of health staff with 10 years’ experience in field of HIV/AIDS, one factor that increases patients’ motivation is providing complementary and free specialized services. The patients stated that if an HIV viral load test is performed in addition to cd4 in every visit and patients are informed of the success of their treatment, they would have higher motivation to continue treatment and visit the centers more. Also, some patients need specialized services such as seeing gynecologists and other specialists and having pap smears and ultrasonography, and they cannot afford the expenses. Therefore, if the services are provided free of charge, they will be motivated to come back. The availability of supplementary medication and supportive packages including food packages in the centers is another factor encouraging patients to visit the centers again.

##### Avoiding provision of specialized health services in specific weekdays and preventing gathering of patients in the BDCCs

One concern raised by patients was the availability of specialists on specific days at some centers which leads to overcrowding and increases the fear of being seen by acquaintances. This can discourage patients from visiting these centers.

*“Sometimes here becomes so crowded, because the specialist comes here just two days a week” (An adolescent male HIV/AIDS patient with a history of 10 years visiting the center)*.

##### Giving patients hope of having a normal life and ensuring them of the effectiveness of the medication and treatment

Health personnel insisted that in first visit when patients face the reality of having HIV/AIDS, they get desperate and become confused. So it is crucial for personnel to revive their hope assure them that a normal and long life is still possible with effective medication and adherence to medical recommendations. Creating such a feeling in patients establishes trust in the quality of the services and increases their adherence to treatment and regular visits to the BDCCs.

### Mutual interactions between personnel and visitors (people living with and at the risk of getting HIV/AIDS)

#### Personnel behavior

##### Not judging and reproaching HIV/AIDS patients and sex workers

Personnel repeatedly emphasized the need to avoid judging or blaming patients during treatment. The focus should be on educating patients about healthy behaviors to improve their overall health and manage their disease, rather than moral judgments. Female sex workers, in particular, expressed fear of being judged during initial visits as a reason for their reluctance to seek care. It is important for personnel with strong religious values to consider whether they are suitable for working in these centers.

Personnel repeatedly emphasized the need to avoid judging or blaming HIV/AIDS patients during treatment. The focus should be on educating patients about healthy behaviors to improve their overall health and manage their disease, rather than reminding them of what is right and what is wrong morally. Female sex workers, in particular, expressed fear of being judged, during the initial visits, as a reason for their reluctance to seek care. This can be challenging for the personnel with strong religious values. It is important for personnel with strong religious values to consider whether they are suitable for working in these centers as it may discourage patients and sex workers from visiting health centers as well.

*“Those who work in Vulnerable Women Counseling Centers, no matter a psychologist or midwife, if they have a judgmental mind, or if they wants to bring their religious values into the work or blame women for what they do, or they look at them badly, the patients will definitely feel it, even if you don’t say anything. In this case, the patients will not come back” (one of the midwifes with 2 years’ work experience in VWCCs)*.

##### Having normal and friendly behavior with HIV/AIDS patients

Establishing a strong staff-patient relationship involves treating patients like normal individuals and avoiding physical distance. Interviewees recommended that it is better minimize the use of masks or gloves during examination and counseling as much as possible while still adhering to safety guidelines, in order to build trust with patients. They may also offer food, sweets, water, and tea to the patients, shake hands with patients of the same sex, place their hands on their shoulders, or even care for their child during counseling or testing. All these behaviors can create a sense of intimacy and trust in patients and reduce the stigma and help them feel that their disease is harmless. Treating patients with respect and kindness is another key components of effective communication.

*“The personnel treat us really well without any expectations, like the way a mother treats her child” (An old male HIV/AIDS patient regularly visiting the BDCC)*.

##### Previous patients satisfied with and trusted in BDCCs and VWCCs as a key factor to assure new cases to visit these centers

The interviewees consistently emphasized that satisfied and trusting current clients play a crucial role in attracting new patients. According to the interviewees, Most of new female sex workers are introduced to VWCCs by their friends and those who have visited the centers before. These peers assure them that there are no threatens and the services provided are completely confidential.

*“The final point that we used is peer group. When someone comes and trusts the center, they realize that there is no judgment here, and health services are provided. They receive education. Then we tell them that we do not have access to others, please introduce this place to your friends, and they bring their friends with them in the next visit. Peer group works very well. When a new person comes, their stress has been already reduced to great extent because of their friends” (A midwife with 8 years’ experience with female sex workers in VWCCs)*.

##### Training the personnel regarding the principles of interaction with HIV/AIDS patients

Interviewees emphasized the importance of providing training to personnel on how to interact with HIV/AIDS patients before they are employed in these centers. These patients require specialized care due to social stigma associated with their disease, their high-risk status, and the need for unique treatments and psychological understanding to establish trust during their initial visit. Unfortunately, many personnel do not possess this knowledge when they begin working and must learn through trial and error.

*“When I first started working in this center, I did not even dare to make any move. I was afraid of the patients. I was really stressed, but after a while, when I saw the kindness between my colleagues and the patients, I felt better, and I changed my attitude” (A midwife with 6 years’ experience in DBCC)*.

##### Getting a better physical and mental feeling in each visit

One of the most recurrent subjects the interviewees pointed out was that the patients should feel better, more cheerful, and more hopeful about their future when they leave the center after each visit. This can highly influence patients’ trust and is one of the responsibilities and issues the personnel should be aware of.

*“One of the doctors talked to me and said there were people who had been living and combating with this disease for almost thirty years, but they had no problems and no one found out about their disease.” (One of regular male HIV/AIDS patients)*.

*“ When they call me, I come here with joy, because when personnel talk to me, I get inspired”. (One of regular old male HIV/AIDS patients)*

##### Wearing a formal uniform

Wearing a uniform and following the principles and dress codes such as wearing name tags were among the issues that some patients mentioned as factors that help them feel secure in these centers. One of the interviewees that it was his first visit to BDCC expressed that he did not trust in female psychologist until she has worn her uniform.

*“The second thing that frightened me was that you were wearing a coat and not a uniform, and you put on a uniform after knowing about my presence. Then, I understood you were an official staff member. You had a name tag and your appearance became professional.”(A young male visitor on his first visit to BDCC)*.

#### Concerns of visitors

##### Patients’ concerns about seeing familiar personnel

In small cities, chances of people knowing each other to see one another in the street or in public places are high, which can become a concern for *HIV/AIDS* patients and sex workers. Therefore, the accent and language of personnel may negatively affect patients’ trust. Patients feel secure more with personnel from other cities because risk of seeing familiar personnel will make them reluctant to return to the centers. However, some of the interviewees stated that most of the patients are from vulnerable groups or sometimes from a particular neighborhood or minority groups, and therefore, know that none of their relatives has a high academic degree or works in public medical centers. Consequently, they are not concerned about the possibility of encountering familiar faces in these centers.

*“Usually, when people go to medical centers, they look for familiar personnel so that they can ask for favors, but in the BDCCs and VWCCs, the clients avoid being seen by their acquaintances or relatives. If we see a patient we know, we should pretend that we did not see anything” (An old male counselor with 6 years’ work experience in BDCCs)*.

##### Concerning about legal problems

A major concerns among HIV/AID patients and especially female sex workers is the fear of legal issues, and potential disclosure of their information to the police or other security institutions. It was also stated that patients feel secure when they know the centers are state-owned and run by the government and that no HIV/AID patients and female sex workers has ever been caught or faced any problem in the past.

*“The first thing that concerns them is the fear that legal and judicial issues may arise, and their personal information may be easily accessed. They worry that they may encounter legal problems. However, we explain to them that the center is a governmental and the government has established this center, and they will not face legal issues. We say that this is just a health center, and so far, no legal problem has arisen. Most of the time, they trust the center with our explanations” (One of personnel with 6 years of working in VWCC)*.

##### Avoiding changing the personnel working in BDCCs and VWCCs

As previously mentioned, one concern for patients, particularly during their initial visits, is the fear of running into familiar faces at the center. Additionally, building trust and intimate relationships between the patient and the personnel takes time. To address these issues, it is recommended that staff members who display good behavior and establish positive communication with the patients should not be transferred unnecessarily. This allows for continuity of care and avoids disrupting the patients’ treatment process. Furthermore, it is important for personnel in these centers to have a close relationship with each other. In crowded centers, if possible, there should be both male and female staff member to provide patients with the option to choose.

*“” Now that this new lady has come to the center, I have to come here at least several times to be able to have a proper conversation with her, but when I talk to you, it’s like I’m talking to my family members.” (One of male HIV/AIDS patients with more than 8 years visiting the center)*.

##### Concerns about taking medications

One of the patients’ concerns is that their disease will be revealed through the drugs they take, so patients have many challenges with taking medicine, and in turn, use different tricks take them secretly. For example, they do not keep the medicines in a special packaging for AIDS medicine, they put them in multivitamin containers or they keep them inside a locked cupboard. Some of the interviewees stated that the high number of drugs used in the past was one of the challenges of regular drug use and increased the possibility of revealing these patients’ diseases. Nowadays, however, with the advancement of medicine and the reduction in the number of medicines used, as well as the reduction of the number of times the medicine is taken during the day, it has become easier for patients to take them secretly.

#### Precautions by visitors

##### Excuses and tricks used by patients and vulnerable women in their first visit

Patients take extreme precautions before their initial visits due to their fear of encountering familiar faces, especially among the personnel. They use many tricks so that if they meet someone they know in the center, they can use a plausible excuse to cover up the main reason for their visit. For example, they ask questions such as what are behavioral diseases? Or I have a friend who has problems in his/her family, can I bring them here? Some also use vaccination, check-ups, or receiving harm-reduction equipment as excuses for their visits. *They might also ask “Does Mr. or Ms. X work here?” or some others come with their friends and act as the companion of another person” (A male psychologist with 5 years’ work experience in BDCCs)*.

##### Using disguise or protective equipment to remain anonymous during visits

The interviewees mentioned that sometimes some patients try to change their appearance as much as possible using things such as sunglasses, face masks, hats, or even veils. This increases their security, and reduce the possibility of being recognized by other familiar patients or personnel.

##### Going to the BDCCs and VWCCs during the last working hours: Spending the minimum possible time in the center

Due to the fact that these patients are always afraid of being seen by the others, when they visit the center, they try to receive services in the shortest possible time and without delay; therefore, they use some tricks. For example, some patients come so late at the end of office hours as they know there are not a lot of people in the center and the personnel are leaving, so that they do not have to spend much time for counseling or retests and the personnel only have time to give them their medications. According to the health personnel, some patients insist on the personnel to get their work done in the center as quickly as possible with excuses such as that the taxi is waiting for them outside.

*“I remember two cases. They brought their husbands to get tested outside office hours.”(A male psychologist with 6 years of working in BDCCs)*.

### Physical characteristics of the centers

#### Location of BDCCs and VWCCs

##### Area of operation: in a quiet or crowded place?

The location of BDCCs and VWCCs is a significant factor that can influence the patient experience. There are differing opinions among patients and personnel regarding whether these centers should be located in quiet, remote areas or in crowded places near other high-traffic organizations such as health centers, hospitals, schools, and banks. Some patients argue that quiet areas are preferable to minimize the likelihood of encountering familiar individuals. On the other hand, some believe that proximity to crowded places, including other health centers, facilitates easier access for patients without explicitly revealing the purpose of their visit. Being close to other health centers allows patients to use getting vaccinated or receiving other health services as a cover for visiting BDCCs and VWCCs. Once they are confident that there are no familiar faces around, they can discuss their real needs. Although the probability of encountering familiar people is obviously higher in these areas, there are more excuses available to conceal the real reason for the visits. However, locating these centers next to or near security organizations such as the police station may instill fear in patients and deter them from seeking care. *“The center used to be in a crowded area. Whenever I was going to BDCC, it was likely to see a few familiar faces, but I made excuses by saying that I am going to the bank, but new BDCC is located in a remote area, so there are few familiar people and less traffic (which is good).” (An adolescent HIV/AIDS patient with regular visits to BDCC)*.

##### Working as independent and separate BDCCs and VWCCs or as a part of a larger building?

Another factor that can significantly impacts patients’ feelings of fear and worry regarding their visits to the BDCCs and VWCCs is whether these centers should operate independently or as part of a larger health center. Interviewees expressed varying opinions on this matter. Some believed that the integrating these centers within other health centers would negatively affect getting routine public health services by ordinary people. This is because some of HIV/AIDS patients have an appearance that is perceived as untidy and disheveled, which could discourage other people from seeking their routine health services. Furthermore, some stated that the integration would increase the likelihood of encountering familiar personnel or people seeking routine health services, as they had personally experienced such encounters. However, most of the interviewees believed that having an independent DBCC or VWCC from the outset brings stigma. If people are seen going to these centers, there will be no excuse to conceal their visits. They argued that integrating these centers into a larger health center with the same entrance, allows patients or female sex workers to conceal the true reason for their visits, making them more comfortable visiting the centers. It was also suggested that having separate entrance and exist in different sides of the building can help HIV/AIDS patients and sex workers to reduce the risk of encountering familiar individuals.

*“I strongly disagree (with integration of BDCCs in health centers) because some patients carry knives with them, some of them are clearly drug addicts, or have an extremely unpleasant appearance, which frightens other patients… HIV/AIDS patients are even opposed to installing CCTV cameras in the center, let alone merging it with a health center” (One of the public health experts with about 5 years’ experience in HIV/AIDS)*.

It should be noted that the State Welfare Organization (SWO) manages the supportive services provided for poor, isolated, and marginalized groups and families with handicapped members. This organization runs centers for training and supporting vulnerable groups including *HIV/AIDS* patients called Positive Clubs. Since a wide range of people with various needs refer to these centers and most of them are healthy, it appears that the integration of BDCCs and VWCCs into the SWO as a supportive institution rather than a medical center could encourage HIV/AIDS patients to visit these centers compared to the integration of these centers into health centers.

*“One of the advantages of our center (Welfare Organization Counseling Center) is that it has the title “Welfare Organization” on its signboard. A lot of visitors including healthy people come here… its signboard doesn’t have a negative connotation and doesn’t make anyone suspicious.”(A counselor working in the Welfare Organization with more than 15 years’ experience in HIV/AIDS)*.

##### Establishing fixed centers and not changing the location of BDCCs and VWCCs

Since the majority of patients refuse to give their identity and contact information, the relocation of the BDCCs and VWCCs, especially if the centers are rented and not owned, can strongly affect the number of patients and sex workers. According to the interviewees, each relocation leads to a significant reduction in the number of patients and sex workers, and therefore, their treatment will be disrupted.

*“If the building of BDCCs and WCCs is owned by the medical university, there would be no relocation, which is great. It is not easy to encourage vulnerable women to come to the center, and now if we relocated, exactly two-thirds of the cases and maybe even more would be lost.”(One of the psychologists with more than 6 years of work in VWCCs)*.

##### The physical space (size) of the BDCCs and VWCCs

The interviewees also highlighted the importance of the physical space and the physical layout of the centers and patients and female sex workers’ fear of encountering acquaintances as factors affecting their willingness to visit the centers. Patients prefer to receive services with minimal contact with others. Therefore, the physical space of the center can either hinder or facilitate visiting the centers. Overall, the majority of interviewees criticized the small physical space of the centers and the potential issues it could cause. Some of the personnel mentioned difficulties in storing a large number of files due to increasingly number of clients. Having a larger space would reduce the chances of encountering other patients, and private areas could be designated for those who wish to wait or conceal themselves from familiar patients.

*“If we had more space and knew that for example, Ms. X is sensitive, we would ask her to wait in a separate room and when it was less crowded, we would visit her privately, but that’s not possible now.” (One of midwives with 8 years of work in VWCCs)*.

*“There were one or two cases who were relatives, and because of not having enough space the unavoidable encounters are increasing among them, which is causing concerns.” (One of psychologists with 2 years’ work experience in VWCCs)*.

*“Sometimes, when I see someone I know from afar, I hide so that they don’t see me and they don’t get anxious, but it’s not possible for all patients because of this small space.”(A female general practitioner with 3 years’ work in BDCCs)*.

#### Geographical distribution of BDCCs and VWCCs

##### Physical distance between BDCCs and VWCCs and living place of people

According to the interviewees, since most of the clients are from vulnerable groups, it is very important to distribute the centers in such a way that they are closest to the patients’ places of residence so that they can find the centers easily and do not have to spend too much for transportation expenses.

##### Not having a catchment area and no geographical division for BDCCs and VWCCs

One of the current benefits of BDCCs and VWCCs is that there is no catchment area and the population of the city is not divided between them based on geographical distribution, and any patient from any part of the city can go to any center in which they feel more secure and less likely to be seen by their relatives, friends, family members, colleagues and other familiar people.

*“People who live near here (*BDCC number 2) *can visit our center if they’d like to, even those who live near* BDCC number*1 but know that they have relatives in that area and aren’t willing to go to that center can visit our center too.” (One of female personnel with 4 years’ working in BDCC number 2)*.

##### Increasing the number of BDCCs and VWCCs

Another factor that can greatly affect patients’ trust and encourage them to visit is increasing the number of these centers in the city. This, especially in bigger cities, would not only make it more convenient for patients to access the centers due to easier access, but it also allow them to choose a center in which they are less concerned about being seen by acquaintances.

#### Physical evidence

##### The signboard of the BDCCs and VWCCs

Different opinions were expressed regarding the appropriateness or the repulsion caused by the title written on the signboard of these centers. Some stated that the title “Behavioral Disorders Counseling Center” is a neutral title because there is no mention of the words HIV or AIDS, and at the same time, it is not very ambiguous, and people in need of services can figure out what services these centers provide and get the services they need. On the other hand, some others found this title repulsive and believed that the title itself specifies the reason for the visits and a more neutral title should be sought.

*“The title of these centers is inappropriate. My niece told me that from the title ‘Behavioral Disorders Counseling Center’ she understood that I was ill.”(A young male HIV/AIDS patient on his first visit)*.

##### Not having surveillance cameras installed

One of the key issues greatly affecting patients’ willingness to visit these centers is that they are very sensitive about the CCTV, which can make them paranoid and afraid of referring to the BDCCs and VWCCs.

*“Because of the equipment here, several times it was suggested that CCTVs should be installed, but it was rejected each time because seeing them can negatively affect patients’ trust” (A male counselor with 5 years’ experience in DBCCs)*.

*“Women keep asking if there are any CCTVs here and we have to reassure them that there are not” (one of the midwives with two years’ experience in VWCCs)*.

##### Not using signs of HIV/AIDS in the BDCCs

To maintain confidentiality and ensure patients’ peace of mind, it was suggested to avoid hanging banners or educational announcements containing HIV/AIDS content in the centers. Some patients even recommended that the lab kits used in the centers should not have the name AIDS or any sign indicating that the majority of clients are HIV/AIDS patients.

To maintain confidentiality and ensure patients’ peace of mind, it was suggested to avoid hanging banners or educational announcements containing HIV/AIDS content in these centers. Some patients even recommended that the lab kits used in the centers should not have the name AIDS and any sign indicating that the majority of clients are HIV/AIDS patients.

“There is no brochure or any sign of HIV/AIDS on the walls in the center” (*A consultant working in counseling centers affiliated with the Welfare Organization with 5 years’ experience with HIV/AIDS patients)*.

*“It was written on the laboratory kits “KITS FOR DIAGNOSING HIV/AIDS”. It should not be like that” (A young male HIV/AIDS patient with regular visits to BDCC)*.

## Discussion

This study investigated the most important factors affecting HIV/AIDS patients and vulnerable women visiting BDCCs and VWCCs respectively. The factors were categorized into three categories including medical and operational processes, mutual interactions between the personnel and visitors (people living with and at the risk of getting HIV/AIDS), and the physical characteristics of the centers. This section provides a discussion of the findings.

Due to the significant social stigma associated with HIV/AIDS in Iran in general and in Ilam and Kermanshah in particular, it is crucial for BDCCs and VWCCs to carefully consider the clinical processes and the provision of medical and counseling services to these patients. The success of the treatment process relies on providing services that meet the clients’ needs and are based on their preferences. The findings of the study indicate that within the current context of Iran, it is important to avoid asking too many questions or focusing on the cause of the disease during the initial visits in order to increase the patient’s interest in continuing their visits to the centers and adhering to the medical instructions. Accordingly, the personnel should not insist on receiving identity information, at least in the first visits, and it can be postponed until trust is established. Using codes instead of personal information can help patients to feel secure, particularly in small towns where familiarity is more likely.

Confidentiality is of utmost importance in BDCCs and VWCCs and the building design should provide a safe environment for clients to feel secure about their privacy. Colleagues should also avoid interrupting the counseling process and create a comfortable atmosphere for all clients. Similarly, studies from Ghana and Sub-Saharan Africa showed that pregnant women and other groups stated doing HIV test in an open and not private space and not trusting health workers’ being confidential as the reasons for refuting taking HIV test [25, 26]. Moreover, studies of the Global Fund to Fight AIDS, Tuberculosis and Malaria showed lack of confidentiality and gossiping as common behaviors among healthcare workers. These behaviors can make HIV patients and sex workers highly worried about no secrecy of their personal information, their HIV status, or sexual activities in the society [27].

Moreover, to address concerns about answering questions regarding the cause and history of visitors’ disease, personnel should wear a uniform with a tag displaying their first and last name and position. Additionally, according to the interviews, some patients may prefer to avoid in-person visits, especially in small provinces, to minimize the chance of encountering familiar faces. Another factor contributing to creation of a positive view of BDCCs is the management of fear of death among HIV/AIDS patients and helping them hope the possibility of living a normal life through regular taking medication. Remote services, such as electronic services and phone calls, can be developed to minimize in-person visits. These services can allow patients to confirm that no familiar or new individuals are present at the centers while visiting the centers. Other relevant studies also indicate that deterioration of physical health and/or death of sexual partner or child are among the enabling factors for up-taking HIV test [26].

Phone or virtual counseling can also ensure confidentiality is maintained. Such facilities can enable patients to make sure no familiar person or new person is in the centers before going there. During the time period that this study was conducted, there were not strong remote and friendly-used online or telephone services in the studied centers, for HIV/AIDS patients or female with risky sexual behaviors to rely on. It definitely make it difficult for visitors, especially new cases, to visit the centers. The stigma of being seen at health services has been stated by previous studies as a major barrier to utilizing health services by HIV patients [28]. To improve health services utilization for HIV/AIDS patients in Iran, it may be useful to create a website with information about the responsibilities, exact address, contact information, and personnel working in the BDCCs. This may reduce patients’ concerns and contribute to more convenient visits, especially, during their first visits. In this regard, social media can also be utilized to promote the website and availability of such centers.

Another crucial factor improving the quality of health services in BDCCs and VWCCs is the employment of personnel who are flexible, patient, and accessible, with effective communicative skills and specialized counseling knowledge for individuals living with or at risk of HIV/AIDS. The personnel should treat clients with dignity and respect, convey medical instructions accurately and motivate and assure patients to continue their treatment. Continuous training courses should be provided to maintain their skills, and the personnel should be selected carefully and not transferred frequently. Additionally, according to our findings, it is advisable to avoid employing health forces with strict religious beliefs and religious appearance in BDCCs and especially in VWCCs as their religious beliefs may hinder effective communication, especially with female sex workers. Personnel should avoid making clients feel blamed or shamed behave normally when encountering the HIV/AIDS patients outside of the center. According to the findings from Ghana and San Francisco, women and drug users mentioned that not being treated well by the nurses and snobby attitude of the staff discouraged them from participating in the facilities for HIV testing and counseling [25, 29, 30]. Other studies also referred to lack of training for medical staff on medical ethics as a reason exacerbating the problem [28]. Feelings of blame and shame and confronting stereotypes of HIV are strong among HIV positive and sex workers and can prevent them from coming to health centers at the first step [44]. Personnel should be aware of that and try to reduce these feelings in their patients.

Findings of our study revealed that patients and sex workers in particular are afraid of being prosecuted by security and legal agencies. Other studies shows similar findings, for example drug users were afraid of “punishment and being locked up” if their HIV test was positive [29] and also in Denmark criminalization hinders women who use drugs from access to harm reduction services [27]. An effective factor in gaining other patients’ trust is the satisfaction of the current and previous clients. Therefore, using this capacity can be effective in expanding the services, identifying other patients, and providing services to a larger population in severe need of these services.

The physical properties and location of the centers are also important considerations. Counseling rooms should be soundproofed to ensure patient conversation cannot be overheard. Similar to standards for building rural and urban comprehensive health centers in Iran, a building plan with defined standards should be developed for BDCCs and VWCCs to address all the concerns raised in this study. Furthermore, financial support should be provided at the national and provincial levels to establish an adequate number of these centers across the country. Insufficient space in some centers for providing a safe waiting area, maintaining confidentiality, reducing unnecessary encounters between the clients, and archiving their files was a major concern among interviewees. Establishing larger centers, especially in small cities, can help alleviate these concerns.

Another important factor rooted in the context of research site which affects health care utilization by people is the location of the of the BDCCs and VWCCs and their operation as independent centers or centers integrated into other medical facilities. According to the findings, it is better to establish BDCCs and VWCCs in crowded areas and near other medical centers. This allows HIV/AIDS patients and vulnerable women to visit the BDCCs and VWCCs more conveniently without drawing attention. Also, most of the interviewees expressed a preference for the centers to be integrated into larger facilities or separate buildings within a larger environment with a common entrance, rather than operating as independent centers. This allows patients to blend in with other clients and conceal the reason for their visit. Although this has been considered in the BDCC booklet, the centers in this study operate as a separate buildings, which creates a barrier to patient visits. Although this may adversely affects health services utilization by other groups. In many countries, such as the US, Sweden, New Zealand, India, Lebanon, Mozambique and Zimbabwe, health centers for HIV/AIDS are located in rural and urban areas, and the public and private sectors work together in this field [45, 46]. However, in Iran, situation is different and these centers are located only in cities and are only run by the governmental sector. Cities are preferred as people are less likely to know each other. Even a part of patients prefer not use health services available in their cities and go to other cities due to social stigma and fear of seeing acquaintances. Due to the substantial stigma associated with this disease in Iran, establishing these centers in rural areas is neither recommended nor possible. In order to preserve the confidentiality of HIV/AIDS patients in Iran, and to properly manage the distribution of specialized medications and diagnostic tests for these patient, all diagnostic and treatment measures for HIV/AIDS patients are centralized and provided free of charge in the public sector through the Behavioral Diseases Counseling Centers. The private sector currently only has the responsibility of referring suspected HIV cases to the BDCCs.

Another notable issue to consider is the establishment of BDCCs and VWCCs in easily accessible locations with the aim of reducing transportation expenses for patients, especially those from poor and vulnerable groups. Increasing the number of these centers in various urban regions not only facilitates patients’ visits and reduces their transportation costs, but also provides them with more options to choose from, thereby addressing concerns about encountering acquaintances during visits. Financial difficulties were identified as a barrier to regular visits to BDCCs and VWCCs. To encourage clients to visit these centers more frequently, interviewees mentioned the importance of proposing financial and food support. Additionally, one positive aspect of these centers in Iran is that there are no geographical restrictions, allowing patients to visit any center they feel comfortable with, regardless of their place of residence. According to the findings of the study and considering the current limitations which exist in Ilam and Kermanshah, it is recommended to avoid using HIV and AIDS signs within the facilities. Furthermore, it was also proposed that diversifying services and offering services for a variety of patients such as tuberculosis, diabetes, thalassemia, hypertension, etc., can provide a better environment for HIV/AIDS people to continue their treatment conveniently. Installing educational banners about other diseases outside the centers and on websites, while highlighting the availability of these facilities, may also increase patients’ visits. Similarly, the study by Downing in San Francisco showed that convenience, increasing the number of HIV test sites, free transportation and monetary incentives were among the main structural motivating factors for drug’ users to take HIV test [29]. Removing financial barriers and convenient access to HIV test were among the facilitators helping people take HIV test in Sub-Saharan Africa [26].

## Study limitations

There were limitations in conducting interviews, as the research team was not allowed to interview HIV/AIDS patients or vulnerable women referring to the BDCCs and VWCCs directly and had to rely on health staff working in the centers instead. This may have affected the quality of the interviews, as patients may not have felt comfortable revealing certain challenges or difficulties to health staff. Additionally, some clients were reluctant to be interviewed or have their voices recorded, so the interviewers had to take notes instead. It is also difficult to have deep interviews with patients, especially on general issues like motives or obstacles of health care utilization.

## Conclusion

In order to enhance the performance of BDCCs and VWCCs and encourage regular visits from HIV/AIDS patients, vulnerable women and those at the risk of getting HIV/AIDS, it is crucial for policy makers and public health officials to address the concerns and issues mentioned in this paper. The clinical processes, physical features, and the way personnel treat patients should change and improve in a way that make patients less afraid of coming to the centers and ensure them that everything has been arranged in order to keep their disease confidential. Further studies are required to measure the value and importance of the factors revealed in this study. For example a survey can measure the weight of each factor in terms of creating trust in people living with HIV/AIDS and sex workers about the performance and secrecy principles of the centers. Moreover, different new features can be extracted from the findings which can be applied to run a new project using conjoint analysis and discrete choice experiments to understand the preferences of visitors and targeted population to know what kind of centers they prefer more and what kinds of changes should be made in these centers to fulfill their expectations.

## Data Availability

All data generated during the current study are in Persian and not publicly available as it is not allowed by the Ethics Committee, but would be available from the corresponding author on reasonable request.

## Abbreviations

BDCCs: Behavioral Diseases Counseling Centers
VWCCs: Vulnerable Women’ Counseling Centers
